# The association of intracranial atherosclerosis with cerebral small vessel disease imaging markers: a high-resolution magnetic resonance imaging study

**DOI:** 10.1038/s41598-023-44240-1

**Published:** 2023-10-09

**Authors:** Kang-Li Zhu, Zi-Yang Shang, Bai-jun Liu, Ying Wang, Jing Li, Ben-Qiang Yang, George Ntaios, Hui-Sheng Chen

**Affiliations:** 1 Department of Neurology, General Hospital of Northern Theater Command, Shenyang, People’s Republic of China; 2 Department of Radiology, General Hospital of Northern Theater Command, Shenyang, People’s Republic of China; 3 Department of Internal Medicine, School of Health Sciences, Faculty of Medicine, University of Thessaly, Larissa, Greece

**Keywords:** Cerebrovascular disorders, White matter disease

## Abstract

To evaluate the association of intracranial non-stenotic atherosclerotic plaque with cerebral small vessel disease (CSVD) imaging markers in a CSVD population using 3.0 T high-resolution magnetic resonance imaging (HRMRI), which was validated in embolic stroke of undetermined source (ESUS) cohort. We retrospectively recruited consecutive patients who were diagnosed with CSVD or ESUS from January 2015 to December 2019. All patients underwent intracranial HRMRI to assess intracranial non-stenotic atherosclerotic plaques. Baseline and imaging data were collected and were measured among all patients. Among 153 patients with CSVD, there were 59 with intracranial atherosclerotic plaque (IAP) and 94 with non-IAP, including 36 with intracranial atherosclerotic complicated plaque (IACP). Among 227 ESUS patients, there were 155 with IAP and 72 with non-IAP, including 127 with IACP. In the CSVD population, we found that: (1) CSVD burden was associated with IAP (*p* = 0.036) and IACP (*p* = 0.008); (2) IAP was associated with white matter hyperintensity (51% vs. 34%; *P* = 0.039), and IACP was associated with lacunes (69% vs. 35%; *P* = 0.009) and enlarge perivascular space (69% vs. 39%; *P* = 0.022). A similar association of CSVD imaging markers with IAP or IACP was found in the ESUS population. Furthermore, the association of unilateral IAP or IACP with CSVD imaging markers of ipsilateral hemisphere was identified in the two cohorts. This is the first report that intracranial non-stenotic atherosclerotic plaque, especially complicated plaque, is closely associated with CSVD imaging markers, which provide further evidence for the association of large artery atherosclerosis with CSVD.

## Introduction

The association of carotid atherosclerotic plaque and its vulnerability with cerebral small vessel disease (CSVD) has been widely investigated^1–4^. For example, asymptomatic CSVD was found to be often accompanied by severe carotid artery stenosis^5^; The characteristics of vulnerable carotid plaques coexisted or were associated with CSVD^6^. In addition to carotid artery, some studies also investigated the association of intracranial artery atherosclerosis (either intracranial portion of the carotids or the circle of Willis) with CSVD, but inconsistent results occurred, which may be attributed to the difference and drawback of intracranial artery atherosclerosis measurement methods including intracranial arterial calcification on CT or the number of vessel wall lesions or blood-flow velocity pulse index or arterial distensibility^7–16^. Given the intracranial artery as the close upstream vessel of CSVD, and these inconsistent results, the relationship between intracranial artery atherosclerosis and CSVD deserves further investigation, especially in Asia population who have a higher prevalence of intracranial artery atherosclerosis^17–20^.

With the development of high-resolution magnetic resonance imaging (HRMRI) technology, the intracranial atherosclerotic plaque can be well identified and more information can be obtained compared with traditional techniques. In this context, the current study aimed to assess the relationship between intracranial non-stenotic atherosclerotic plaque and CSVD imaging markers by 3.0 T HRMRI in the CSVD cohort, which was further validated in embolic stroke of undetermined source (ESUS) cohort to confirm the reliability and generalizability of their association given the different etiologies between two cohorts.

## Methods

### Study population

We used the same ESUS and CSVD cohorts, which have been reported in detail in our recent study^21^. Briefly, eligible patients were adults presenting with acute stroke symptom who were diagnosed with unilateral anterior circulation ischemic by DWI, received high resolution magnetic resonance examination of the head within 1 week of onset and met the diagnostic criteria of ESUS and CSVD. In addition, all CSVD patients were diagnosed with arteriolosclerosis origin rather than cerebral amyloid angiopathy. The main exclusion criteria included non-stenosis carotid plaque with ≥ 3 mm thickness; aortic arch atherosclerotic plaque thickness ≥ 4 mm or ulcerative plaque, or history of balloon dilation or stent implantation or bilateral infarction, etc. Patient informed consent was waived given the retrospective nature of the analysis and minimal risk to subjects by the institutional review board of General Hospital of Northern Theater Command. Briefly, between January 2015 and December 2019, we enrolled all consecutive CSVD and ESUS patients at the Department of Neurology of General Hospital of Northern Theater Command. All enrolled patients underwent intracranial HRMRI measurements (including 3D T1-weighted and 2D T2-weighted imaging sequences) to assess the presence and vulnerability of plaques. We collected demographic data, clinical characteristics, a range of laboratory indicators, and neuroimaging data (including Flair T2, etc.). The detailed inclusion/exclusion criteria were shown in supplemental Table S1.

### Imaging protocol

All MRI scans were performed on 3.0 Tesla MRI scanners (GE discovery MR750, Milwaukee, WI) using an 8-channel head coil with the standardized acquisition protocols (including 3-dimensional T1-weighted and 2-dimensional T2-weighted imaging sequences) for multidimensional evaluation of plaque. A fat suppression technique was used to reduce fat signals from the scalp. Zero-filled Fourier transform (ZIP 512, ZIP2) was used to reduce pixel size, and the final display resolution was 0.3 to 0.4 mm3, with a scan range of > 480 mm covering the internal carotid artery, middle cerebral artery, anterior cerebral artery, vertebral artery, basilar artery, and posterior cerebral artery. MRI scanning took 20 to 25 min, which is described in detail elsewhere^21^. In addition, two trained raters (Z.Y.S. and D.W.) with > 2 years of experience reviewing intracranial magnetic resonance images who were blinded to the clinical data analyzed magnetic resonance image quality by consensus, using a previously developed 4-point scale (1 = poor quality, 2 = acceptable, 3 = good quality, 4 = excellent), in which images of poor quality were excluded from our final result. Qualitative and quantitative analyses were performed using ImageJ version 1.49 (National Institutes of Health, Bethesda, Maryland) and RadiAnt DICOM Viewer version 5.0.2 (Medixant, Poznan, Poland) for 3-dimensional volume rendering using the appropriate magnification. The window width and level were adjusted to optimize the conspicuity of vessel contour.

### MRI examination and CSVD burden assessment

Imaging markers of CSVD mainly include lacune presumed to be of vascular origin after excluding other possible reasons, white matter hyperintensity (WMH) presumed to be of vascular origin after excluding other possible reasons, enlarged perivascular space (EPVS), and cerebral microbleed (CMB)^22^. We calculated the number of lacunes, EPVS (EPVSs in the centrum of the oval and the basal ganglia respectively), and CMBs (lobar and deep/ infratentorial CMBs respectively) in both hemispheres of the brain. The EPVS was scored on a 5-grade semi-quantitative scale (0–4: 0 = no EPVS; 1 = 1–10 EPVS; 2 = 11–20 EPVS; 3 = 21–30 EPVS; 4 = 40–EPVS)^23^. The Fazekas scale (range from 0 to 3) was used to rate periventricular and deep WMH^24^. Finally, we calculated the presence of these four neuroimaging markers to determine the total CSVD burden based on an ordinal “CSVD score” (range from 0 to 4) according to previous study^25^. One point was awarded for any of the following definitions: ≥ 1 lacunes, ≥ 1 cerebral microbleeds, moderate to severe (grade 2–4) EPVS in basal ganglia or the centrum of the oval, periventricular WMH Fazekas 3 (extending into deep white matter) and/or deep WMH 2–3 (early confluent).

### Definition of plaque on HRMRI

The signal characteristics of intracranial non-stenotic atherosclerosis plaque (IAP) components were determined as described previously^21^. The intracranial atherosclerosis plaque (IAP) was identified as markedly eccentric or focal wall thickening, with the thickest wall ≥ 50% of the thinnest part on HRMRI^26^. According to the American Heart Association (AHA) definition of type VI plaque, intracranial atherosclerosis complicated plaque (IACP) is defined as any or both discontinuity of plaque surface (DPS) or intraplaque hemorrhage (IPH). IPH is defined as T1 hypersignal greater than 150% of the adjacent muscle or pons. DPS is defined as an irregular luminal surface the of plaque, such as fibrous cap rupture, ulcerated plaque, or mural thrombus^27^. Based on the presence or absence of non-stenotic IAP or IACP (regardless of ipsilateral, contralateral, or bilateral), the patients were divided into IAP vs non-IAP group, and IACP vs non-IACP group, respectively.

### Statistical analysis

Continuous variables were described as mean ± SD (if normally distributed), or median and IQR (if not normally distributed), while categorical variables are expressed as absolute and relative frequencies. We used Student's *T* test or Wilcoxon's rank sum-test for continuous data, and χ^2^ or Fisher’s exact test for categorical data. Demographic, clinical data and serological markers were compared between ESUS and CSVD patients with vs without IAP and with vs without IACP. Multifactorial analyses were performed to adjust for confounding factors such as age, sex, and other vascular risk factors to explore the association between IAP or IACP with CSVD neuroimaging markers. In order to adjust the confounding factors, these variables of vascular risk factors with *P* < 0.1 in binary univariate logistic regression analysis were included in binary multivariate logistic regression analysis. In addition, CSVD neuroimaging markers were rated by 2 researchers, who were blinded to clinical information. Interrater reliability and plaque characteristics was measured by the intraclass correlation coefficient (ICC), which was interpreted as followed: < 0.5: poor; 0.5–0.75: moderate; 0.75–0.9: good; > 0.9: excellent. Analyses were performed with SPSS software (version 26.0; IBM), when *P* < 0.05, the difference was statistically significant.

### Ethics approval

This retrospective study was approved by the Institutional Review Board of General Hospital of Northern Theater Command (IRB: k2019-57).

## Results

### Study population characteristics

From the initial ESUS and CSVD cohorts, we excluded 16 out of 243 and 7 out of 160 patients, respectively, due to poor image quality or incomplete information. Therefore, a total of 380 patients were eventually included in the study including 227 ESUS patients and 153 CSVD patients. Supplemental Table S2 summarizes detailed baseline demographic characteristics and laboratory examination in patients with and without non-stenotic IAP. In the CSVD cohort, older age ([62.78 ± 9.38] vs [59.30 ± 9.45], *P* = 0.038), lower female incidence (20% vs 26%), higher incidence of diabetes and prior stroke or transient ischemic attack, higher glycated hemoglobin, more WMHs, and higher CSVD burden were found in patients with IAP compared to patients without. In the ESUS cohort, older age (62 [56–69] vs 58 [51–66], *p* = 0.014), and lower female incidence (30% vs. 42%), higher incidence of hypertension, higher glycated hemoglobin and cystatin c, more WMHs, lacunae and EPVSs, and higher CSVD burden were found in IAP compared to patients without. Similar demographic and laboratory data, as well as CSVD imaging markers, were found in patients with and without IACP in the two cohorts (Supplemental Table S3).

### Logistic regression analyses for the association between intracranial non-stenotic atherosclerosis plaque and CSVD imaging markers

**Figure 1:**
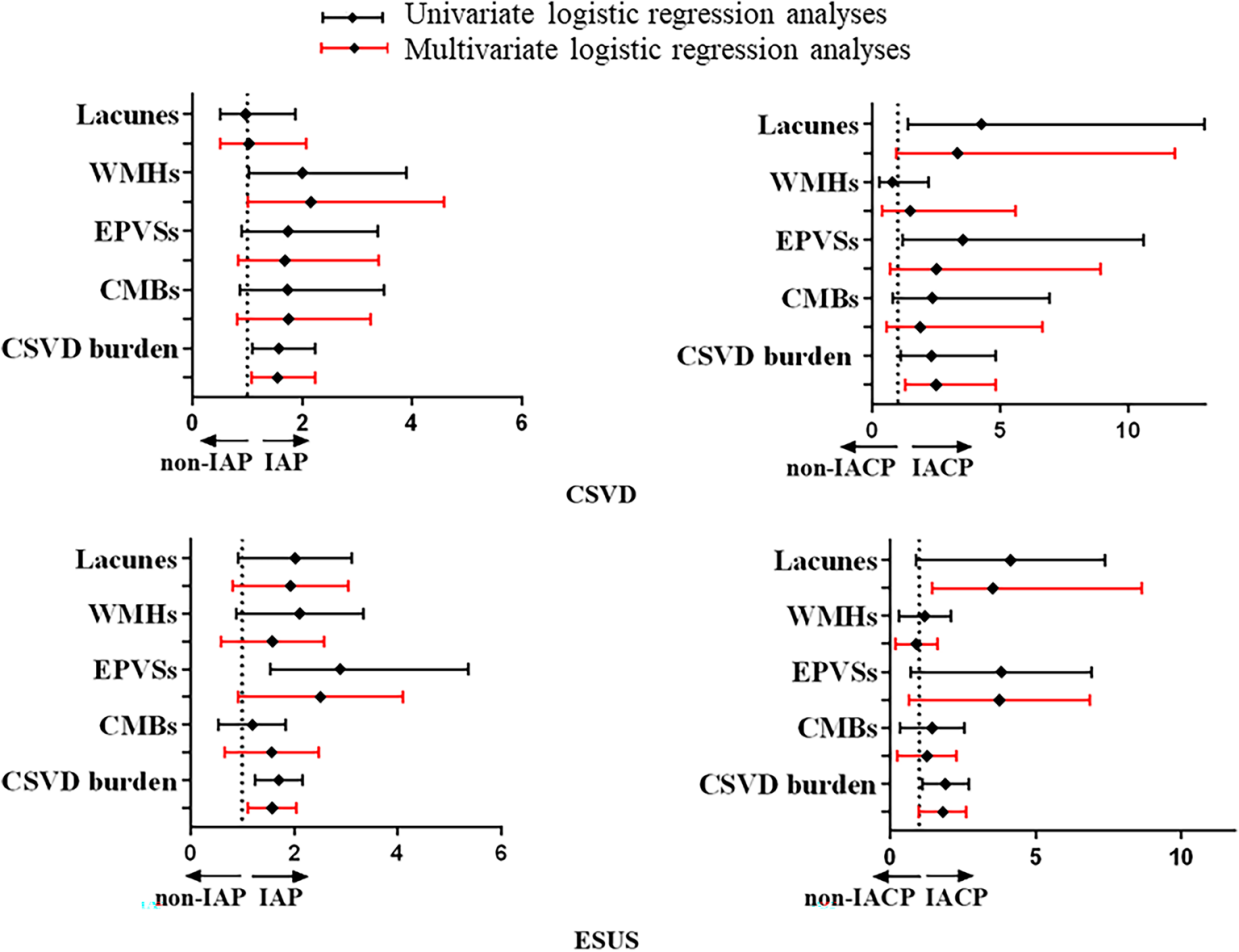
Logistic regression analyses for the association of intracranial atherosclerosis plaque with CSVD imaging markers in CSVD and ESUS patients. CSVD, cerebral small vessel disease; ESUS, embolic stroke of undetermined source; WMH, white matter hyperintensity; EPVS, enlarge perivascular space; CMB, cerebral microbleed; IAP, intracranial atherosclerosis plaque; IACP, intracranial atherosclerosis complicated plaque.

**Figure 2:**
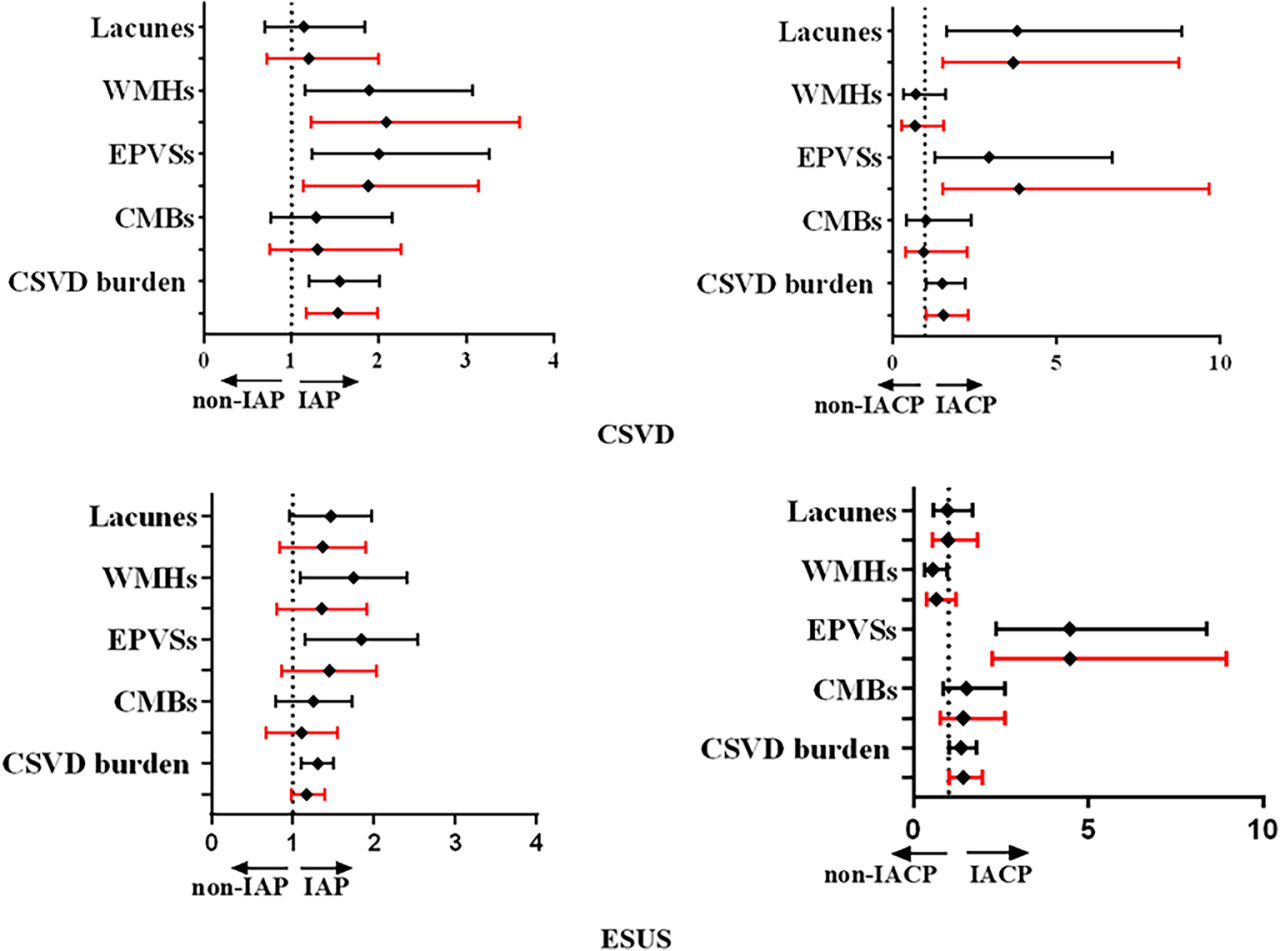
Logistic regression analyses for the association of unilateral intracranial atherosclerosis plaque with ipsilateral hemisphere CSVD imaging markers in CSVD and ESUS patients. Multivariate Logistic regression analyses after adjusted for age, gender, current smoker, alcohol use, hypertension and diabetes mellitus. CSVD, cerebral small vessel disease; ESUS, embolic stroke of undetermined source; WMH, white matter hyperintensity; EPVS, enlarge perivascular space; CMB, cerebral microbleed; IAP, intracranial atherosclerosis plaque; IACP, intracranial atherosclerosis complicated plaque.

In CSVD patients, WMHs (2.00 [1.03–3.90], *P* = 0.041) and CSVD burden (1.57 [1.10–2.23], *P* = 0.012) were associated with IAP, while lacunes (4.26 [1.4–12.97], *P* = 0.011), EPVSs (3.54 [1.18–10.60], *P* = 0.024), and CSVD burden (2.49 [1.28–4.83], *P* = 0.007) were associated with IACP. No relationship between CMBs and plaques was found in the CSVD cohort (Table 1). Similar results were found in the ESUS cohort: lacunes (1.83 [1.04–3.21], *P* = 0.037), WMHs (1.88 [1.02–3.44], *P* = 0.042), EPVSs (2.89 [1.55–5.36], *P* = 0.001) and CSVD burden (1.67 [1.27–2.18], *P* < 0.001) were correlated with IAP, while lacunes (3.28 [1.40–7.71], *P* = 0.006), EPVSs (2.97 [1.22–7.25], *P* = 0.017), and CSVD burden (1.78 [1.15–2.75], *P* = 0.010) were associated with IACP (Table 1). After adjusting for gender, age, smoking, alcohol consumption, hypertension, diabetes, there was still an obvious correlation between CSVD neuroimaging markers and IAP or IACP in the two cohorts (Fig. 1, Table 1). Furthermore, to eliminate potential confounding factors between individuals, we further determined the association of unilateral IAP with ipsilateral hemisphere CSVD imaging markers. The results showed that unilateral IAP was associated with ipsilateral hemisphere CSVD imaging markers (Table 2, Fig. 2). Similar results about the association of CSVD imaging markers with unilateral IACP were identified in the two cohorts (Table 2, Fig. 2).

**Table 1:** Logistic regression analyses for the association of intracranial atherosclerosis plaque with imaging markers of CSVD.

	CSVD				ESUS			
	IAP vs non-IAP (59 vs. 94)		IACP vs non-IACP (36 vs. 23)		IAP vs non-IAP (155 vs. 72)		IACP vs non-IACP (127 vs. 28)	
	OR (95% CI)	*p* value	OR (95% CI)	*p* value	OR (95% CI)	*p* value	OR (95% CI)	*p* value
Lacunes								
Univariable	0.97 (0.51–1.87)	0.938	4.26 (1.40–12.97)	0.011	1.83 (1.04–3.21)	0.037	3.28 (1.40–7.71)	0.006
Multivariable	1.03 (0.50–2.08)	0.932	3.33 (0.94–11.83) 0	.063	1.71 (0.93–3.14)	0.083	3.54 (1.45–8.63)	0.006
WMHs								
Univariable	2.00 (1.03–3.90)	0.041	0.77 (0.27–2.20)	0.625	1.88 (1.02–3.44)	0.042	0.96 (0.42–2.18)	0.913
Multivariable	2.16 (1.02–4.58)	0.044	1.48 (0.39–5.59)	0.564	1.37 (0.71–2.66)	0.354	0.69 (0.28–1.71)	0.422
CMBs								
Univariable	1.73 (0.86–3.50)	0.124	2.34 (0.80–6.91)	0.122	1.07 (0.61–1.89)	0.812	1.16 (0.50–2.65)	0.733
Multivariable	1.75 (0.82–3.25)	0.149	1.88 (0.54–6.63)	0.324	1.39 (0.76–2.55)	0.286	0.99 (0.41–2.36)	0.974
EPVS								
Univariable	1.74 (0.90–3.37)	0.099	3.54 (1.18–10.60)	0.024	2.89 (1.55–5.36)	0.001	2.97 (1.22–7.25)	0.017
Multivariable	1.68 (0.83–3.39)	0.148	2.50 (0.70–8.92)	0.158	2.18 (1.12–4.24)	0.022	2.88 (1.15–7.21)	0.024
CSVD burden								
Univariable	1.57 (1.10–2.23)	0.012	2.49 (1.28–4.83)	0.007	1.67 (1.27–2.18)	< 0.001	1.78 (1.15–2.75)	0.010
Multivariable	1.55 (1.08–2.24)	0.019	2.32 (1.12–4.82)	0.024	1.53 (1.13–2.07)	0.007	1.68 (1.06–2.68)	0.029

*CSVD* cerebral small vessel disease, *ESUS* embolic stroke of undetermined source, *WMHs* white matter hyperintensities, *CMBs* cerebral microbleeds, *EPVSs* enlarge perivascular spaces, *IAP* intracranial atherosclerotic plaque, *IACP* intracranial atherosclerotic complicated plaque. Multivariable logistic regression analyses: adjusting for gender, age, smoking, alcohol consumption, hypertension, and diabetes.

**Table 2:** Logistic regression analyses for the association of unilateral intracranial atherosclerosis plaque with ipsilateral hemisphere CSVD imaging markers.

	CSVD				ESUS			
	IAP vs non-IAP (98 vs 208)		IACP vs non-IACP (53 vs 45)		IAP vs non-IAP ( 242 vs 212)		IACP vs non-IACP (169 vs 73)	
	OR (95% CI)	*p* value	OR (95% CI)	*p* value	OR (95% CI)	*p* value	OR (95% CI)	*p* value
Lacunes								
Univariable	1.14 (0.70–1.84)	0.603	3.81 (1.64–8.83)	0.002	1.42 (0.98–2.00)	0.066	0.97 (0.56–1.69)	0.916
Multivariable	1.20 (0.72–2.00)	0.497	3.69 (1.55–8.76)	0.003	1.30 (0.87–1.93)	0.196	0.99 (0.54–1.82)	0.970
WMHs								
Univariable	1.89 (1.16–3.07)	0.011	0.72 (0.33–1.61)	0.427	1.66 (1.13–2.44)	0.010	0.55 (0.32–0.96)	0.035
Multivariable	2.09 (1.22–3.61)	0.008	0.68 (0.30–1.57)	0.373	1.28 (0.84–1.95)	0.259	0.66 (0.36–1.22)	0.188
CMBs								
Univariable	1.28 (0.77–2.15)	0.345	1.03 (0.44–2.39)	0.984	1.20 (0.82–1.75)	0.347	1.51 (0.87–2.63)	0.144
Multivariable	1.30 (0.75–2.26)	0.357	0.96 (0.40–2.28)	0.916	1.05 (0.70–1.58)	0.800	1.42 (0.77–2.63)	0.263
EPVSs								
Univariable	2.00 (1.23–3.26)	0.005	2.95 (1.29–6.72)	0.010	1.76 (1.20–2.58)	0.004	4.47 (2.37–8.40)	** < **0.001
Multivariable	1.88 (1.13–3.14)	0.015	3.86 (1.54–9.67)	0.004	1.37 (0.91–2.07)	0.135	4.48 (2.24–8.96)	** < **0.001
CSVD burden								
Univariable	1.55 (1.20–2.01)	0.001	1.52 (1.04–2.23)	0.030	1.30 (1.11–1.51)	0.001	1.36 (1.02–1.81)	0.038
Multivariable	1.53 (1.17–1.99)	0.002	1.56 (1.04–2.33)	0.031	1.17 (0.98–1.39)	0.081	1.43 (1.03–1.97)	0.033

*CSVD* cerebral small vessel disease, *ESUS* embolic stroke of undetermined source, *WMHs* white matter hyperintensities, *CMBs* cerebral microbleeds, *EPVSs* enlarge perivascular spaces, *IAP* intracranial atherosclerotic plaque, *IACP* intracranial atherosclerotic complicated plaque.

Multivariable logistic regression analyses: adjusting for gender, age, smoking, alcohol consumption, hypertension, and diabetes.

To further confirm the relationship between intracranial atherosclerotic plaque and CSVD burden, we dichotomized the overall CSVD burden severity as none to mild (score 0–2) and moderate to severe (score 3–4). In the CSVD cohort, moderate-to-severe CSVD burden was more prevalent in the patients with IAP vs non-IAP (25.43% vs 8.51%, *P* = 0.004, Table 3) or IACP vs non-IACP (41.66% vs 13%, *P* = 0.02, Table 3). IACP was found to have the highest proportion of moderate-to-severe CSVD burden, followed by the patients with IAP and finally the patients with non-IAP (41.66% vs. 25.43% vs. 8.51%, Table 3). Similar results were also found in ESUS patients (Table 3).

**Table 3:** Analyses for the association of intracranial atherosclerotic plaque with CSVD burden.

CSVD burden n (%)	Non-IAP (n = 94)	IAP (n = 59)	*p* value	Non-IACP (n = 23)	IACP (n = 36)	p value
CSVD						
0–2 score	86 (91.49)	44 (74.57)	0.004	20 (87.00)	21 (58.34)	0.020
3–4 score	8 (8.51)	15 (25.43)		3 (13.00)	15 (41.66)	

	Non-IAP (n = 72)	IAP (n = 155)	p value	Non-IACP (n = 28)	IACP (n = 127)	*p* value
ESUS						
0–2 score	57 (79.16)	98 (63.22)	0.026	25 (89.30)	77 (60.63)	0.002
3–4 score	15 (20.84)	57 (36.78)		3 (10.70)	50 (39.37)	

*CSVD* cerebral small vessel disease, *ESUS* embolic stroke of undetermined source, *IAP* intracranial atherosclerotic plaque, *IACP* intracranial atherosclerotic complicated plaque.

### Reproducibility

In addition, interrater reliability was good for the measurement of CSVD biomarkers: the ICC of lacunes, WMHs, CMBs, and EPVSs was calculated as 0.80, 0.85, 0.73, and 0.78, respectively. Similarly, the ICC of IAP and IACP was calculated as 0.85 and 0.87, respectively.

## Discussion

In this HRMRI-based analysis, we assessed the association of non-stenotic IAP with CSVD imaging markers and identified a relationship between CSVD and IAP/IACP in both CSVD and ESUS patients. Furthermore, the reliability of this correlation is also supported by our finding of an association between unilateral IAP/IACP and ipsilateral hemisphere CSVD imaging markers in both cohorts, which significantly limits potential confounding factors between individuals.

CSVD burden can reflect brain microstructural damage, because the presence of CSVD often indicates impaired cerebrovascular reserve^28,29^. Given that cerebral large arterial segments like the carotid arteries represent the upstream of cerebral small vessel and they share common vascular risk factors^30–32^, the association between carotid artery atherosclerosis and CSVD has been widely investigated and well recognized^1–6,33,34^. Given that the intracranial arterial segments lie closer to the small cerebral vessels than the carotid artery, it could be hypothesized that intracranial atherosclerosis may be more closely associated with CSVD. In the current study, we found that non-stenotic IAP was closely associated with CSVD imaging markers in both CSVD and ESUS cohorts. The findings provided further evidence for the association of large artery atherosclerosis with CSVD. Previous studies also investigated the association of intracranial atherosclerosis with CSVD, but the results were conflicting, which may be attributed to imperfect measurements or other potential confounding factors. For example, some studies assessed intracranial atherosclerosis by calculating the volume and severity of intracranial arterial calcification, but one main limitation of this approach is that calcification on CT is only a fraction of IAP, and thus cannot accurately reflect IAP^9–11,35,36^. Some studies used intracranial atherosclerosis burden (defined as the number of vessel wall lesions) to determine the relationship between intracranial atherosclerosis and CSVD^7,8^. In addition, in some studies, blood-flow velocity pulse index and arterial distensibility were used as indicator to explore the correlation between intracranial atherosclerosis and CSVD^12,37,38^. In the current study, we used IAP, identified by HRMRI, as an imaging marker of intracranial artery atherosclerosis, which can provide more information of intracranial atherosclerosis compared with these previous methods. Previous studies showed that carotid vulnerable plaque is related to CSVD burden^4,6,39,40^. Given that complicated plaques may represent active artery atherosclerosis, there is reason to believe that such plaques may be more likely associated with the cerebral small vessel. Indeed, in the current study, we found a strong association of IACP with CSVD, which further supports the relationship between large artery atherosclerosis and CSVD. In addition, we found no relationship between CMBs and IAP, which is consistent with previous studies^8,41^. This seems in concordance with the previous view that microbleeds are generally caused by intrinsic small vessel disease, such as hypertensive vasculopathy and cerebral amyloid angiopathy^42^.

The main strength of this study is that it is the first to investigate the association of non-stenotic IAP and IACP with CSVD imaging markers based on HRMRI, and provide evidence for their association. Second, for the first time, our study identified their relationship in non-stenotic intracranial arteries, in contrast to previous studies which explored the relationship between large artery atherosclerosis and CSVD in stenotic vessels (either the extracranial or the intracranial artery). In addition, we demonstrated the relationship of unilateral non-stenotic IAP or IACP to ipsilateral hemisphere CSVD imaging markers, which could limit the potential confounders, given that more prevalence of intracranial atherosclerotic plaque or complicated plaques ipsilateral to stroke^21,43^. Finally, the association of non-stenotic IAP or IACP with CSVD imaging markers was demonstrated in both cohorts, which further supports the reliability of the conclusion.

We acknowledge some limitations. First, this is a retrospective analysis, which might be subject to selection bias. To eliminate potential confounders, we assessed also the association of unilateral IAP or IACP with ipsilateral hemisphere CSVD imaging markers, and the results support their association. Second, the moderate sample size is another limitation, but our strict inclusion and exclusion criteria might enhance the reliability of the conclusion. Third, lack of histological validation on the plaque is a common limitation in HRMRI studies, although preliminary studies have demonstrated a high level of agreement between MRI-defined plaque signal features and histology^8,21^. Fourth, given the young age, relatively low percentages of risk factors, the exclusion of more than > 50% ICAS stenosis, and the relatively low mean CVSD burden in current population, these findings limits the implication in a higher-risk population. Fifth, there was no measurement of plaque volume or degree of stenosis in the current study. In addition, CSVD markers only including WMHs, EPVS, CMBs, and lacunes is incomplete due to inclusion of recent small subcortical infarcts and brain atrophy. Sixth, contrast enhancement was not used in the current cohort, given that contrast enhancement of atherosclerotic plaque is a strong, reliable marker of culprit plaques^40^. Seventh, we only investigated the association in CSVD and ESUS populations due to lack of HR-MRI data in other stroke subtypes. In addition, ESUS patients with bilateral infarctions and non-stenosis carotid plaque with ≥ 3 mm thickness were excluded, which cannot represent usual ESUS patients. These limitations may affect the generalization of this finding. Finally, given the high prevalence of intracranial atherosclerosis in Asia population^17–20^, the relationship between non-stenotic IAP and CSVD imaging markers needs to be confirmed also in other non-Chinese populations.

## Conclusion

In this HRMRI-based analysis, we found that intracranial non-stenotic atherosclerotic plaque, especially complicated plaque, is closely associated with CSVD imaging markers. These findings provide evidence to support the association between cerebral large artery atherosclerosis and CSVD.

### Supplementary Information

Supplementary Information.

## Data Availability

The data underlying this article will be shared upon reasonable request to the corresponding author.
