# Supplementary material for: The association of intracranial atherosclerosis with cerebral small vessel disease imaging markers: a high-resolution magnetic resonance imaging study
Source: Sci Rep. 2023 Oct 9;13:17017. doi: 10.1038/s41598-023-44240-1 (PMC10562462; doi:10.1038/s41598-023-44240-1)
Supplement: Supplementary file 1 — Supplementary Information. [file 41598_2023_44240_MOESM1_ESM.docx]

**Supplemental Table 1. The detailed inclusion/exclusion criteria of CSVD and ESUS**

| ESUS | CSVD |
| --- | --- |
| Inclusion criteria |  |
| 1. Age >18 years | 1. Age >18 years |
| 2. Acute ischemic stroke in the territory of unilateral anterior circulation (DWI lesion and corresponding acute neurological deficit of >24 h in duration) | 2. Acute ischemic stroke in the territory of unilateral anterior circulation (DWI lesion and corresponding acute neurological deficit of >24 h in duration) |
| 3. Within 1 week of onset to high-resolution MRI | 3. Within 1 week of onset to high-resolution MRI |
| 4. Fulfillment of diagnostic criteria for ESUS | 4. Symptom caused by lacunar infarction (defined as a subcortical infarct ≤2 cm on DWI in largest dimension) |
| 4a. Stroke detected by DWI that was not lacunar (defined as a subcortical infarct ≤2 cm on DWI in largest dimension) | 5. Absence of extracranial or intracranial atherosclerosis causing ≥50% luminal stenosis in arteries supplying the area of ischemia |
| 4b. Absence of extracranial or intracranial atherosclerosis causing ≥50% luminal stenosis in arteries supplying the area of ischemia | 6. No major-risk cardioembolic source |
| 4c. No major-risk cardioembolic source | 7. No other specific cause of stroke identified (e.g., arteritis, dissection, migraine/vasospasm, drug abuse) |
| 4d. No other specific cause of stroke identified (e.g., arteritis, dissection, migraine/vasospasm, drug abuse) |  |
| Exclusion criteria |  |
| 1. Nonstenotic carotid plaque ≥ 3 mm in thickness detected on CTA or carotid ultrasonography | 1. Nonstenotic carotid plaque ≥ 3 mm in thickness detected on carotid ultrasonography ( |
| 2. Aortic arch atherosclerotic plaque with ulceration or $4 mm in thickness on CTA or TEE | 2. Bilateral infarcts on DWI |
| 3. Patients with balloon dilatation and stent | 3. Previous radiation therapy to head or neck |
| 4. Bilateral infarcts on DWI | 4. Malignant tumor |
| 5. Previous radiation therapy to head or neck |  |
| 6. Malignant tumor |  |

CTA=computed tomographic angiography; DWI=diffusion-weighted imaging; ESUS=embolic stroke of undetermined source; MRI=magnetic resonance imaging; CSVD = cerebral small vessel disease; TEE=transesophageal echocardiography.

**Supplemental Table 1. Comparison of demographic characteristics and laboratory examination on in patients with IAP vs non-IAP.**

|  | CSVD |  |  | ESUS |  |  |
| --- | --- | --- | --- | --- | --- | --- |
|  | IAP (n=59) | non-IAP (n=94) | p value | IAP (n=155) | non-IAP (n=72) | p value |
| Age, years | 62.78±9.38 | 59.30±9.45 | 0.038 | 62 (56,69) | 58 (51,66) | 0.014 |
| Gender (female), n (%) | 12 (20) | 24 (26) | 0.461 | 47 (30) | 30 (42) | 0.093 |
| Current smoker, n (%) | 35 (59) | 50 (53) | 0.458 | 72 (47) | 29 (40) | 0.384 |
| Alcohol use, n (%) | 31 (53) | 43 (46) | 0.413 | 61 (39) | 31 (43) | 0.597 |
| Hypertension, n (%) | 43 (73) | 56 (60) | 0.094 | 91 (59) | 27 (38) | 0.003 |
| Diabetes mellitus, n (%) | 22 (37) | 15 (16) | 0.003 | 41 (27) | 11 (15) | 0.062 |
| CAD, n (%) | 3 (5) | 5 (5) | 0.949 | 22 (14) | 4 (6) | 0.057 |
| Prior stroke or TIA, n (%) | 19 (32) | 13 (14) | 0.007 | 36 (23) | 11 (15) | 0.169 |
| Initial NIHSS | 2 (1-4) | 2 (1-3) | 0.226 | 4 (1-7) | 5 (1-8) | 0.315 |
| Hyperlipidaemia | 46 (78) | 65 (69) | 0.234 | 79 (51) | 33 (46) | 0.468 |
| WBC, 10^9/L | 7.0 (6.2-8.1) | 7.5 (6.20-8.60) | 0.381 | 8.2 (6.6,10.5) | 7.5 (6.78,8.34) | 0.021 |
| HB, g/L | 144 (132-154) | 141.5 (131-158) | 0.634 | 143 (128.156) | 141 (129,150) | 0.355 |
| PLT, 10^9/L | 210 (176-252) | 218 (185.8-267.5) | 0.085 | 219 (192,265) | 230 (198,270) | 0.301 |
| HbA1c, % | 6.30 (5.50-6.60) | 5.90 (5.58-6.30) | 0.047 | 5.80 (5.60-6.30) | 5.65 (5.43-6.00) | 0.045 |
| HCY, umol/L | 12.50 (9.68-16.52) | 10.19 (8.78-14.60) | 0.121 | 11.66 (9.40-15.61) | 11.43 (8.69-14.96) | 0.445 |
| Ur, mmol/L | 5.68 (4.76-6.85) | 5.82 (4.56-7.10) | 0.756 | 5.21 (4.47-6.46) | 5.04 (3.88-5.73) | 0.083 |
| Cr, umol/L | 69.67 (62.00-81.91) | 72 (60.80-87.63) | 0.802 | 68.10 (55.74-79.20) | 65.47 (56.42-76.06) | 0.327 |
| CysC, mg/L | 0.86 (0.74-1.01) | 0.89 (0.74-1.10) | 0.433 | 0.90 (0.71-1.05) | 0.86 (0.70-0.93) | 0.016 |
| UA, umol/L | 315 (266-363) | 316 (247-399.5) | 0.885 | 305 (263-379) | 305 (265.5-346) | 0.830 |
| Lacunes, n (%) | 31 (53) | 50 (53) | 0.938 | 92 (59) | 32 (44) | 0.036 |
| WMHs, n (%) | 30 (51) | 32 (34) | 0.039 | 65 (42) | 20 (28) | 0.040 |
| CMBs, n (%) | 22 (37) | 24 (26) | 0.123 | 93 (60) | 36 (50) | 0.157 |
| EPVSs, n (%) | 30 (51) | 35 (37) | 0.097 | 76 (49) | 18 (25) | 0.001 |
| CSVD burden | 2 (1-3) | 2 (1-2) | 0.036 | 2 (1-3) | 1 (1-2) | **＜**0.001 |

CAD=Coronary artery disease; TIA=Transient ischemic attack; WBC=White blood cell; PLT=Platelet; HbA1c=Glycosylated hemoglobin; HCY=Homocysteine; Ur=Serum urea; Cr=Creatinine; CysC=Cystatin C; UA=Uric acid; CSVD=Cerebral small vessel disease; ESUS= Embolic stroke of undetermined source; WMHs=White matter hyperintensities; CMBs=Cerebral microbleeds; EPVSs=Enlarge perivascular spaces; IAP=Intracranial atherosclerotic plaque. Values are presented as mean ± SD, median (interquartile range), or frequencies (%).

**Supplemental Table 2．Comparison of demographic characteristics and laboratory examinati****on in patients with IACP vs non-IACP.**

|  | CSVD |  |  | ESUS |  |  |
| --- | --- | --- | --- | --- | --- | --- |
|  | IACP (n=36) | non-IACP (n=23) | p value | IACP (n=127) | non-IACP (n=28) | p value |
| Age, years | 62.14±8.96 | 63.78±10.12 | 0.530 | 64 (60-71) | 47 (40-51) | **＜**0.001 |
| Gender (female), n (%) | 4 (11) | 8 (35) | 0.028 | 35 (38) | 12 (43) | 0.111 |
| Current smoker, n (%) | 25 (69) | 10 (44) | 0.048 | 56 (44) | 16 (57) | 0.210 |
| Alcohol use, n (%) | 23 (64) | 8 (35) | 0.029 | 45 (35) | 16 (57) | 0.033 |
| Hypertension, n (%) | 22 (61) | 21 (91) | 0.011 | 77 (61) | 14 (50) | 0.301 |
| Diabetes mellitus, n (%) | 14 (39) | 8 (35) | 0.750 | 36 (28) | 5 (18) | 0.255 |
| CAD, n (%) | 1 (3) | 2 (9) | 0.313 | 21 (17) | 1 (4) | 0.075 |
| Prior stroke or TIA, n (%) | 8 (22) | 11 (48) | 0.040 | 31 (24) | 5 (18) | 0.457 |
| Initial NIHSS | 2 (1-4) | 2 (2-4) | 0.881 | 3 (1-7) | 4 (1-8) | 0.147 |
| Hyperlipidaemia | 23 (64) | 4 (17) | 0.704 | 66 (52) | 12 (44) | 0.467 |
| WBC, 10^9/L | 7.0 (5.98-7.78) | 7.0 (6.60-8.20) | 0.658 | 7.90 (6.40,9.48) | 7.80 (5.90,10.5) | 0.715 |
| HB, g/L | 145.5 (132-155.5) | 137.0 (132.0-152.0) | 0.316 | 143 (129,155) | 139 (124,156) | 0.243 |
| PLT, 10^9/L | 197.0 (167.25-240.5) | 216.0 (184.0-257.0) | 0.347 | 221 (193,262) | 230 (195,270) | 0.666 |
| HbA1c, % | 12.56 (8.70-16.72) | 11.88 (10.45-16.13) | 0.858 | 5.80 (5.60-6.50) | 5.70 (5.49-6.08) | 0.168 |
| HCY, umol/L | 197 (167-240) | 216 (184-257) | 0.950 | 11.65 (9.66-15.70) | 11.98 (8.75-14.35) | 0.706 |
| Ur, mmol/L | 5.58 (4.82-6.64) | 5.98 (4.11-7.15) | 0.926 | 5.25 (4.56-6.58) | 5.18 (4.10-5.94) | 0.323 |
| Cr, umol/L | 72.81 (64.48-80.00) | 67.27 (49.90-85.29) | 0.539 | 68.17 (55.0-79.08) | 67.75 (60.46-79.82) | 0.326 |
| CysC, mg/L | 0.86 (0.70-1.04) | 0.85 (0.77-1.01) | 0.732 | 0.93 (0.75-1.08) | 0.76 (0.67-0.89) | 0.002 |
| UA, umol/L | 327.5 (270.5-385.5) | 276.0 (237.0-344.0) | 0.063 | 293 (261-362) | 394 (297.5-471.5) | **＜**0.001 |
| Lacunes, n (%) | 25 (69) | 8 (35) | 0.009 | 82 (65) | 10 (36) | 0.005 |
| WMHs, n (%) | 18 (50) | 13 (57) | 0.625 | 53 (42) | 12 (43) | 0.913 |
| CMBs, n (%) | 20 (56) | 8 (35) | 0.119 | 77 (61) | 16 (57) | 0.733 |
| EPVSs, n (%) | 25 (69) | 9 (39) | 0.022 | 69 (54) | 8 (29) | 0.014 |
| CSVD burden | 2 (2-3) | 2 (1-2) | 0.008 | 2 (1-3) | 2 (1-2) | 0.006 |

CAD=Coronary artery disease; TIA=Transient ischemic attack; WBC=White blood cell; PLT=Platelet; HbA1c=Glycosylated hemoglobin; HCY=Homocysteine; Ur=Serum urea; Cr=Creatinine; CysC=Cystatin C; UA=Uric acid; CSVD=Cerebral small vessel disease; ESUS= Embolic stroke of undetermined source; WMHs=White matter hyperintensities; CMBs=Cerebral microbleeds; EPVSs=Enlarge perivascular spaces; IACP= Intracranial atherosclerotic complicated plaque. Values are presented as mean ± SD, median (interquartile range), or frequencies (%).
